# Phylogenetic and Functional Diversity of Microbial Communities Associated with Subsurface Sediments of the Sonora Margin, Guaymas Basin

**DOI:** 10.1371/journal.pone.0104427

**Published:** 2014-08-06

**Authors:** Adrien Vigneron, Perrine Cruaud, Erwan G. Roussel, Patricia Pignet, Jean-Claude Caprais, Nolwenn Callac, Maria-Cristina Ciobanu, Anne Godfroy, Barry A. Cragg, John R. Parkes, Joy D. Van Nostrand, Zhili He, Jizhong Zhou, Laurent Toffin

**Affiliations:** 1 Ifremer, Laboratoire de Microbiologie des Environnements Extrêmes, UMR6197, ZI de la pointe du Diable, Plouzané, France; 2 Université de Bretagne Occidentale, Laboratoire de Microbiologie des Environnements Extrêmes, UMR6197, ZI de la pointe du Diable, Plouzané, France; 3 CNRS, Laboratoire de Microbiologie des Environnements Extrêmes, UMR6197, ZI de la pointe du Diable, Plouzané, France; 4 Ifremer, Laboratoire Etude des Environnements Profonds, UMR6197, ZI de la pointe du Diable, Plouzané, France; 5 Université de Brest, Domaines Océaniques IUEM, UMR6538, Place Nicolas Copernic, Plouzané, France; 6 Ifremer, Géosciences Marines, Laboratoire des Environnements Sédimentaires, ZI de la pointe du Diable, Plouzané, France; 7 School of Earth and Ocean Sciences, Cardiff University, Cardiff, United Kingdom; 8 Institute for Environmental Genomics and Department of Microbiology and Plant Biology, University of Oklahoma, Norman, Oklahoma, United States of America; 9 State Key Joint Laboratory of Environment Simulation and Pollution Control, School of Environment, Tsinghua University, Beijing, China; 10 Earth Science Division, Lawrence Berkeley National Laboratory, Berkeley, California, United States of America; Argonne National Laboratory, United States of America

## Abstract

Subsurface sediments of the Sonora Margin (Guaymas Basin), located in proximity of active cold seep sites were explored. The taxonomic and functional diversity of bacterial and archaeal communities were investigated from 1 to 10 meters below the seafloor. Microbial community structure and abundance and distribution of dominant populations were assessed using complementary molecular approaches (Ribosomal Intergenic Spacer Analysis, 16S rRNA libraries and quantitative PCR with an extensive primers set) and correlated to comprehensive geochemical data. Moreover the metabolic potentials and functional traits of the microbial community were also identified using the GeoChip functional gene microarray and metabolic rates. The active microbial community structure in the Sonora Margin sediments was related to deep subsurface ecosystems (Marine Benthic Groups B and D, Miscellaneous Crenarchaeotal Group, *Chloroflexi* and Candidate divisions) and remained relatively similar throughout the sediment section, despite defined biogeochemical gradients. However, relative abundances of bacterial and archaeal dominant lineages were significantly correlated with organic carbon quantity and origin. Consistently, metabolic pathways for the degradation and assimilation of this organic carbon as well as genetic potentials for the transformation of detrital organic matters, hydrocarbons and recalcitrant substrates were detected, suggesting that chemoorganotrophic microorganisms may dominate the microbial community of the Sonora Margin subsurface sediments.

## Introduction

Deep marine subsurface sediments are one of the most extensive microbial habitats on Earth, covering more than two-thirds of the Earth's surface and reaching maximal thickness of more than 10 km at some locations [1]. Microbial populations are widespread in these sediments as deep as temperature permits [2] and cell numbers vary consistently ranging from 10^10^ to 10^3^ cells per cm^3^ of sediments according to their proximity from land, sedimentary rates and depth [3]. In general, microbial abundance in subsurface sediments (below 1 mbsf) decreases exponentially with depth, as a probable consequence of the decreasing organic carbon quality and availability [4]. Recent investigations based on NanoSIMS monitoring [5] or intact ribosomal RNA [6] and membrane lipid detection [6], [7] demonstrate that sedimentary microbial communities are active as they can incorporate carbon and nitrogen. However, overall metabolic rates are very slow, with biomass turnovers ranging from years to millennia [8]. Numerous of studies have focused on elucidating the microbial diversity of subsurface sediments [6], [9]–[13]. Specific lineages of *Bacteria* (for e.g. *Chloroflexi*, Candidate division JS1) and *Archaea* (for e.g. Miscellaneous Crenarchaeotal Group (MCG), Marine Benthic Group D (MBGD), South African Goldmine Euryarchaeotal Group (SAGMEG) [14], [15], distinct from the surface biospheres (above 1 mbsf), appear to occur consistently in marine subsurface sediments. However identification of the metabolism of these microbial populations remains challenging. Isotopic signatures of membrane lipids suggested that heterotrophic strategies dominated in these ecosystems [6], [7]. Metagenomic and metatranscriptomic analyzes of subsurface sediments from the deep biosphere of the Peru Margin revealed metabolisms associated with lipids, carbohydrates and amino acids utilization. However detected genes and transcripts were mainly affiliated to *Firmicutes*, *Actinobacteria*, and *Alpha*- and *Gammaproteobacteria* rather than *Archaea*, *Chloroflexi* and candidate divisions [16], [17]. Finally, recent single cell genomic approaches indicated the capacity of peptides degradation for members of MCG and MBGD archaeal lineages [18]. Despite these recent advances the metabolic pathways associated to the dominant microbial communities in subsurface sediments remain unclear.

The cold seeps of the Sonora Margin in the Guaymas Basin (Gulf of California), colonized by visible microbial mats and faunal assemblages, were previously characterized as highly active areas with abundant concentrations of methane and sulfur cycle microorganisms (Anaerobic methanotrophs, sulfate-reducing bacteria) in the shallow sediments (0–20 cmbsf) [19], [20]. However, the subsurface microbial communities and processes that occur in the deeper sediments of the Sonora Margin have not yet been explored. The aim of this study was therefore to estimate the phylogenetic and functional biodiversity of the Sonora Margin sediments by comparing the geochemical composition, the microbial taxonomic diversity and abundance, and the GeoChip-based metagenome from subsurface sediments sampled in proximity with active cold seeps of the Sonora Margin. We analyzed the archaeal and bacterial diversity, abundance and distribution in correlation with geochemical gradients and elementary composition of the sediments and compared with the Sonora Margin surface cold seep sediments. Furthermore, we identified the metabolic processes and the functional potentials in term of carbon utilization and energy for both bacterial and archaeal communities and present insights into the microorganism adaptability and capacity to use various substrates in marine subsurface sediments.

## Materials and Methods

### Core sampling and abiotic variables

Sediment samples were collected from Sonora Margin cold seeps in the Guaymas Basin, during the Ifremer “BIG” cruise on the research vessel *L'Atalante* in June 2010. This cruise has benefited from a work permit in Mexican waters by the Mexican Secretariat of Foreign Relations (DAPA/2/281009/3803, October 28th, 2009). Gravity core BCK1 (N 27°35.804, W 111°28.697), 10 meters in length, was recovered from an observed gas depression in methane plume fields, 600 meters distant from visible active cold seeps (WM14 and EWM14 in Vasconcelos area [20]), at 1723 meters water depth. *In situ* temperatures, measured using thermal sensors (THP, Micrel) attached to the core, increased gradually from 3.5°C at the water-sediment interface to 5°C in the bottom of the core (9 mbsf). Immediately after retrieval, BCK1 core was sectioned in 1 meter long sections and transferred into the cold room. The plastic core liner was opened every 50 cm for sub-sampling. Samples for molecular analysis were collected aseptically using cut-off sterile 5 mL syringes, and frozen at −80°C. Sediment samples for activity rate estimations were taken using five cut-off sterile 5 mL syringes per section. These syringes were hermetically and anaerobically sealed with nitrogen in aluminum bags (Grüber-Folien, Germany) and stored at 4°C for processing back to laboratory. Methanogenic activity measurements from Acetate, Di-methylamines and CO_2_ substrates were carried out at Cardiff University, UK, as detailed in Methods S1.

Pore water was obtained by spinning down approximately 10 grams of crude sediment then was fixed as previously described [20]. Sulfate concentrations were determined by ion exchange chromatography as previously described [21]. Hydrogen sulfide and ammonium concentrations were measured by colorimetry [22]. Methane concentrations were quantified using the headspace technique (HSS Dani 86.50) and a gas chromatograph (Perichrom 2100) equipped with a flame-ionization detector [23]. Total organic carbon (TOC) of the sediments were measured by combustion in a LECO CS 125 carbon analyzer, as previously detailed [24]. Quantitative elemental chemical compositions of unfiltered pore waters were measured using Inductively Coupled Plasma-Atomic Emission Spectrophotometry (ICP-AES, Ultima 2, Horiba, JobinYvon), as previously detailed [25]. Effect of eventual particle contaminations was limited by normalization of the elemental concentrations by conservative element (Na) concentrations. The stable-isotope composition of methane was measured by ISOLAB b.v. company (Neerijen, The Netherlands) in the first meter deep section (0.5 mbsf) and in the deepest sediment layer (8.5 mbsf) as previously described [20].

### Nucleic acids extraction and amplifications

Total nucleic acids (DNA and RNA) were directly extracted in duplicate from 2.5 grams of sediments [26], then pooled and purified [27]. Total RNA was purified from crude nucleic acids using Nucleospin RNA II Kit (Macherey Nagel, Düren, Germany) prior to RT-PCR. Aliquots of rRNA were reverse transcribed using Quanta qScript kit according to manufacturer's protocol (Quanta Bioscience, Gaithersburg, MD, USA). As control for DNA contamination, no amplification was obtained by PCR on RNA aliquots. All molecular experiments were carried out as previously monitored in surface cold seep sediments of the Sonora Margin [20]. PCR primers and appropriate annealing temperatures are listed in Table S1. Sequencing of 16S rRNA transcripts and their analysis including, taxonomic affiliations and phylogenetic trees were performed as detailed in Methods S1. Automated ribosomal intergenic spacer analysis (ARISA) of the archaeal and bacterial communities and real-time (q)PCR experiments targeting various sedimentary microbial lineages (*Archaea*, ANME-1, ANME-2a, ANME-2c, ANME-3, Methanosarcinales, Methanomicrobiales, Methanococcales, Methanobacteriales, Methanopyrales, MCG, MBGB, MBGD, *Bacteria*, *Chloroflexi*, Candidate division JS1, *Desulfosarcina*/*Desulfococcus*, *Desulfobulbus*, SEEP SRB2; Table S1) were carried out on purified DNA samples every 50 cm from 1 mbsf to 9 mbsf as presented in Methods S1. Statistical tests were carried out using the software PAST [28]. Nucleic acid sequences are available in the EMBL database under the following accession numbers: HF543837–HF543861 for archaeal, HF545450–HF545524 for bacterial 16S rRNA sequences and HF935025–HF935037 for *mcrA* gene sequences.

### GeoChip analysis

The GeoChip 4.0 microarray, containing 83992 oligonucleotide probes and targeting 152414 gene variants in 401 categories for different microbial functional and biogeochemical processes was monitored as previously detailed [29]. Although the GeoChip was initially based on the genome of cultured microorganisms, the new generation of GeoChip has been extensively enriched with metagenome data from various environments and contains now an important number of relevant probes targeting genes from cultured and uncultured microorganisms involved in key biogeochemical cycles. Total purified DNA samples were labeled then hybridized on GeoChip slides. Signal intensities were scanned and spots with signal-to-noise ratios lower than 2 were removed before analyses [29]. The phylogenetic design of the data acquisition enabled confident assignment of metabolic capabilities to bacterial and archaeal phyla [30], [31], thus dataset were sorted according to the taxonomic affiliation of the genes (*Bacteria*, *Euryarchaeota* and *Crenarchaeota*). Output was analyzed using the GeoChip 4.0 data analysis pipeline [32] and tested using the statistical software PAST [28]. Relative signal intensity was normalized by the number of the probes for each indicated metabolic pathway. List of targeted genes for each category are provided in Table S2. Visualization of the bacterial and archaeal functional potential was achieved using spider dendrograms, where each arm of the plot corresponded to a metabolic pathway. The raw GeoChip dataset is available at http://ieg.ou.edu/4download/.

## Results

### Geochemical description

**Figure 1:**
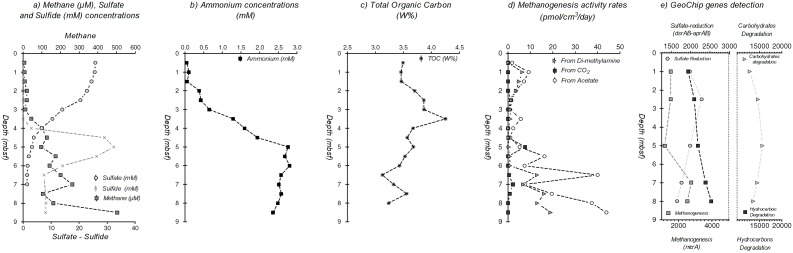
Geochemical depth profiles, putative methanogenesis activity rates and GeoChip genes detection of the sediment core BCK1. 1a) Dissolved methane (grey square, µM), sulfate (white circle, mM) and sulfide (grey cross, mM) concentrations in pore waters. 1b) Dissolved ammonium concentrations (mM) in pore waters. 1c) Total organic carbon (TOC) content in the sediments (% w/w). 1d) Methanogenesis activity rates from acetate (white circle), bicarbonate (black square) and di-methylamine (grey triangle) in the sediments (pmol/cm^3^/day). 1e) Relative signal intensity of the GeoChip microarray for sulfate-reduction (circle), methanogenesis (grey square), carbohydrates degradation (triangle) and hydrocarbon degradation (black square) pathways, normalized by the number of the probes for each indicated metabolic pathway.

The BCK1 core, from the Sonora Margin sediments, showed the typical geochemical signatures of continental margin sediments (Figure 1a), with a sulfate to methane transition zone (SMTZ) located around 5 mbsf. Sulfate pore water concentrations decreased from 25 mM at the sediment-water interface down to 2 mM at 5.5 mbsf. Hydrogen sulfide concentrations were only detected in the deeper sediment layers with a maximum of 32 mM at 5 mbsf decreasing to around 8 mM at 8 mbsf. Methane pore water concentrations increased with depth reaching 500 µM at the bottom of the sediment core (8.5 mbsf). and were positively correlated with the methanogenesis rates (Pearson correlation coefficient *r* = 0.72, *P* = 0.001; <45 pmol/cm^3^/d at 8.5 mbsf) (Figure 1d). Isotopic signature of methane was −97.3‰ at the bottom of the core (9 mbsf) and −82‰ at 1 mbsf, confirming that most of methane produced was from biogenic origin and indicating that methane oxidation potentially occurred towards the sediment surface. Ammonium concentrations, likely resulting of organic matter degradation, increased with depth until reaching 2.5 mM at 5 mbsf (Figure1b). Total organic carbon (TOC) content varied between 3.1 and 4.3% (w/w) throughout the sediment with peaks at 3.5, 5 and 7.5 mbsf (Figure 1c). Analysis of the element composition of the pore water highlighted both a manganese reduction zone in the first meter of sediment and specific horizons (3.5, 5–6, 7 and 8 mbsf) with significant enrichment of metallic elements (Fe, Al, Si, Mn, Ti) (Figure S1). These increases of metal concentrations in pore water suggest detrital terrigenous inputs in the sediment layers, as previously detected in the Guaymas Basin [33].

### Microbial community structure and composition

**Figure 2:**
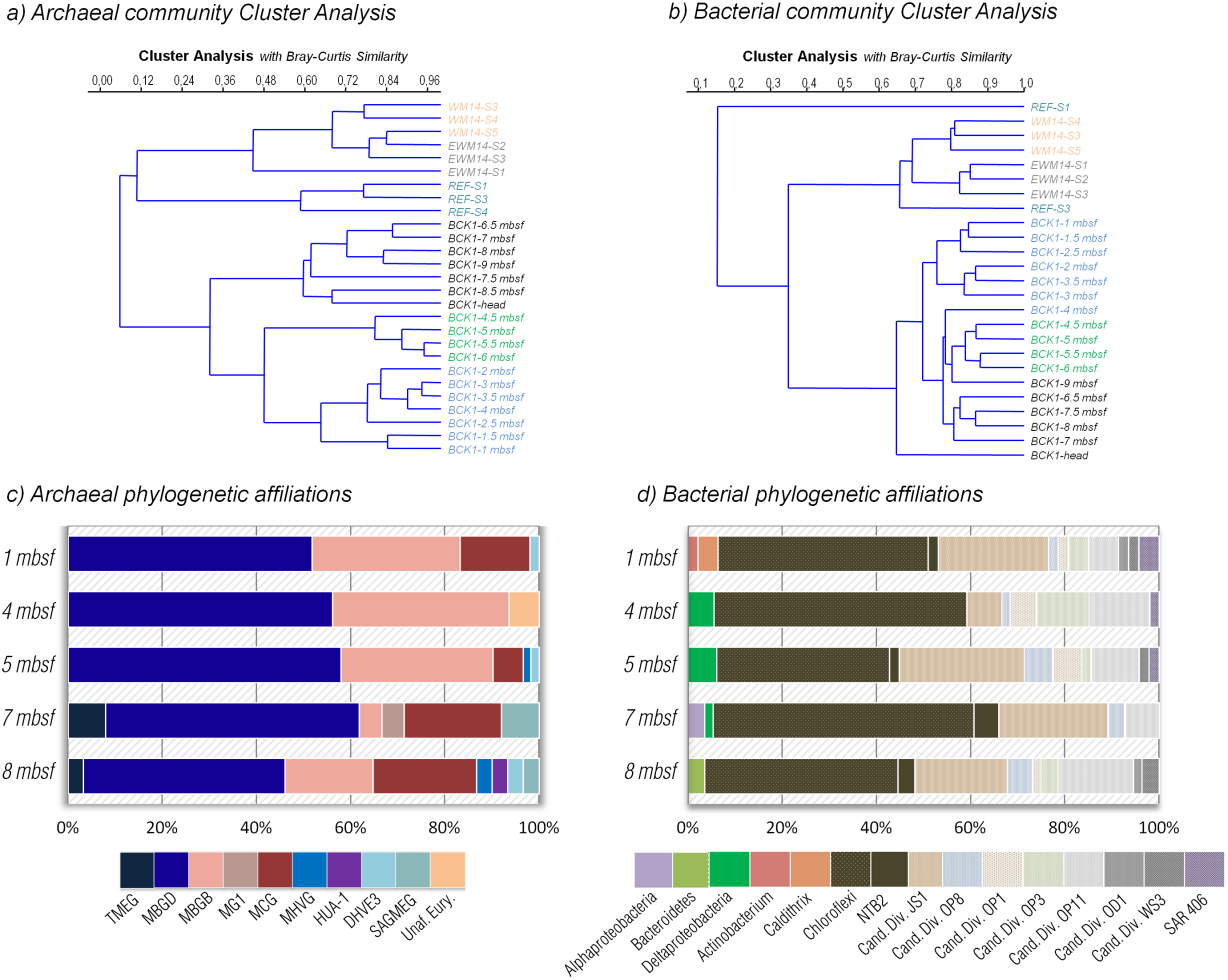
Microbial diversity. Clustering analyses using unweighted pair-group average (UPGMA) and Bray-Curtis Similarity measure of the a) archaeal and b) bacterial community structures visualizing the ARISA dataset. Depth distribution of the c) archaeal and d) bacterial phylogenetic affiliations of the 16S rRNA-derived sequences at 1, 4, 5, 7 and 8 mbsf sediment layers of BCK1. WM14 mbsf sediment layers of BCK1. WM14 (White Microbial mat), EWM14 (Edge of White Microbial mat) and REF (reference outside active seepage area) samples were previously analyzed with the same material and method in Vigneron et al 2013 and corresponded to archaeal community structure of the surface sediments of the Sonora Margin. TMEG, Terrestrial Miscellaneous Euryarcheotal Group; MBGD/B, Marine Benthic Group D/B; MG I, Marine Group I; MCG, Miscellaneous Crenarchaeotic Group; MHVG, Marine Hydrothermal Vent Group; Hua1, Huasco archaeal group 1; DHVE3, Deep-Sea Hydrothermal Vent Euryarchaeotal Group 3; SAGMEG, South Africa Gold Mine Euryarchaeotal Group.

Microbial community structure variations with depth were compared from the Sonora Margin cold seep surface sediments using ARISA. The archaeal and bacterial community structures of the BCK1 were significantly different from the surface sediments of both cold seep (WM14 and EWM14 samples [20]) and outside active seepage areas (REF samples [20]) as shown by clustering and ANOSIM (p<0.0008) on ARISA dataset (Figure 2) Dendrogram and Nonmetric Multidimensional Scaling (NMDS) analysis, based on Bray-Curtis similarity measure also indicated that BCK1 samples clustered according to sediment depths (1–4 mbsf, 4.5–6 mbsf and 6.5–9 mbsf). However this observation was not statistically supported by ANOSIM and seemed to rather reflect a difference in signal intensity more than in community composition.

Based on geochemical features, representative sediment depth horizons (1, 4, 5, 7 and 8 mbsf) were selected for the 16S rRNA survey. A total of 565 partial 16S rRNA sequences (303 for Archaea and 262 for Bacteria) were obtained and used as a proxy for active microbial communities [34]–[36]. Overall, statistical analysis of the microbial community structure of the samples indicated that the microbial community was nearly constant throughout the sediment core (SIMPER average similarities between paired samples above 74.05%).

Archaeal 16S rRNA libraries showed a very limited diversity throughout the sediment core (1-H*_Simpson_* = 0.615±0.08; Figure 2, Figure S2, Figure S3), including three uncultivated phylotypes, mainly found in the deep biosphere: the Marine Benthic Groups B and D (MBGB, MBGD) and the Miscellaneous Crenarchaeotal Group (MCG), mainly represented by the MCG-8 and MCG-10 sub-groups [37]. Other groups such as South Africa Gold Mine Euryarchaeotal Group (SAGMEG), Marine Hydrothermal Vent Group (MHVG) and Terrestrial Miscellaneous Euryaechaeotal group (TMEG) were also detected in lower proportions in the deepest sediment layers.

In contrast, the bacterial 16S rRNA libraries indicated a larger diversity (1-H*_Simpson_* = 0.712±0.06), dominated by *Chloroflexi* and diverse bacterial candidate divisions including JS1, OP11, OP1, OP8 and OP3 (Figure 2, Figure S2, Figure S4). The *Chloroflexi* lineage included different sub-groups and most of the amplified sequences were relatives to *Dehalococcoidetes* or subphylum IV groups. A few *Deltaproteobacteria*, usually related to sulfate-reducers and hydrocarbon degraders in cold seep sediments were detected in 4, 5 and 7 meters depth sediment horizons.

### Microbial 16S rRNA gene abundance and distributions

**Figure 3:**
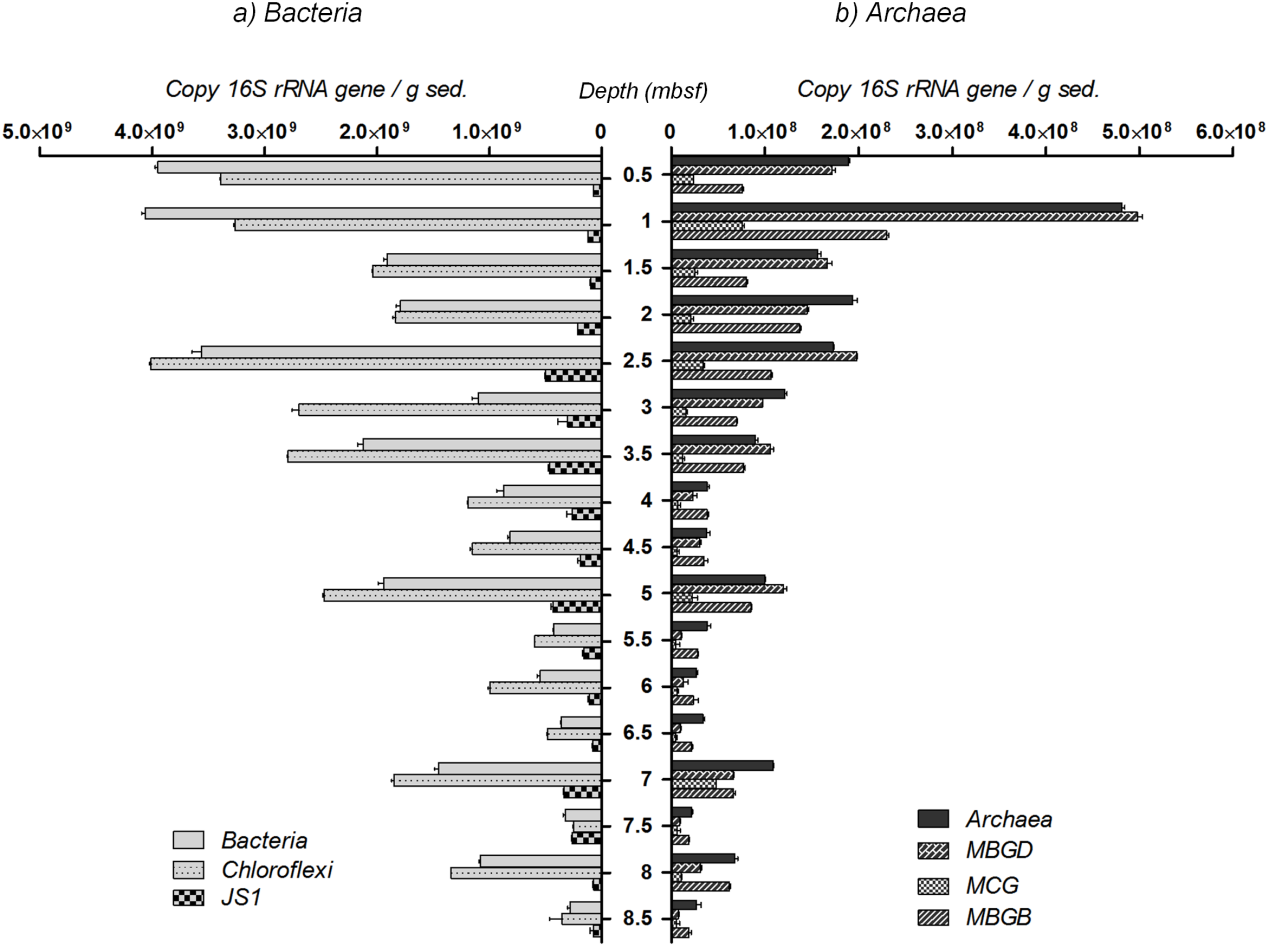
Q-PCR estimations. Q-PCR estimation of 16S rRNA gene copy numbers per gram of sediment for a) total *Bacteria* and bacterial groups of *Chloroflexi*, candidate division JS1 and b) total *Archaea* and archaeal groups of Marine Benthic Group B (MBGB), D (MBGD), Miscellaneous Crenarchaeotal Group (MCG), from BCK1 sediment core. Methanosarcinales were only detected at 1 mbsf with 2.4 mbsf with 2.4×10^6^ 16S rRNA gene copies g^−1^ but were not represented in the figure. ANaerobic MEthanotrophs (ANME), *Desulfosarcina*/*Desulfococcus* (DSS), *Desulfobulbus* (DBB) and other methanogens orders were not detected in analyzed samples.

Depth distributions and relative abundance of microorganisms were analyzed every 50 cm by real-time PCR (Figure 3). 16S rRNA gene abundance of *Bacteria* was around 10 fold higher than *Archaea* throughout the sediment core, and decreased with depth from 4×10^9^ 16S rRNA gene copies per gram of sediment in the top of the core to 2.8×10^8^ copies at the bottom. Bacterial relative abundance showed elevated concentrations in particular at 5, 7 and 8 meters below the seafloor with 1.95×10^9^, 1.45×10^9^ and 1.1×10^9^ 16S rRNA gene copies g^−1^ respectively. As sequences affiliated to *Chloroflexi* and candidate division JS1 dominated bacterial 16S rRNA gene libraries, the 16S rRNA genes of these groups were specifically quantified. *Chloroflexi* 16S rRNA gene abundance was estimated by subtracting JS1 16S rRNA gene copy numbers from quantifications with JS1 and *Chloroflexi* groups specific primers [38]. *Chloroflexi* 16S rRNA gene abundance appeared to mirror the bacterial distribution profile (Pearson correlation coefficient *r* = 0.914, *P*<0.0001) and strongly dominated the bacterial community throughout the sediment core. In contrast, JS1 16S rRNA gene copy numbers increased with depth until reaching maximum values between 2.5 and 5 mbsf with 4.62×10^8^ copies g^−1^. No cold seep sulfate-reducing bacteria (*Desulfosarcina/Desulfococcus* and *Desulfobulbus* groups) were detected.

Total archaeal 16S rRNA gene copy numbers, represented 4–10% of the total number of 16S rRNA gene and decreased with depth, from 1.9×10^8^ 16S rRNA gene copies g^−1^ at 1 mbsf to 2.67×10^7^ 16S rRNA gene copies at the bottom of the sediment core. However, specific horizons (1 mbsf, 5 mbsf, 7 and 8 mbsf) showed peaks of elevated archaeal 16S rRNA gene concentrations with 4.8×10^8^, 1×10^8^, 1.1×10^8^ and 6.8×10^7^ 16S rRNA gene copies respectively. Within the *Archaea*, uncultivated groups MBGD, MBGB and MCG were detected throughout the sediment core. Their distributions were correlated with the archaeal distribution (Pearson correlation coefficient *r* = 0.98, *P*<0.0001) and no specific niche repartition was detected along the sulfate and methane concentration gradients. Assuming the same 16S rRNA copy number for each microbial lineage, MCG were fivefold less abundant than marine benthic groups except at 7 mbsf with 4.8×10^7^ 16S rRNA gene copies g^−1^. Consistently with 16S rRNA library results, ANME lineages were below the detection limit (<10^4^ 16S rRNA gene copies g^−1^) and methanogens were only represented by Methanosarcinales at 1 mbsf with 2.4×10^6^ 16S rRNA gene copies g^−1^.

### Functional gene diversity and GeoChip array

**Figure 4:**
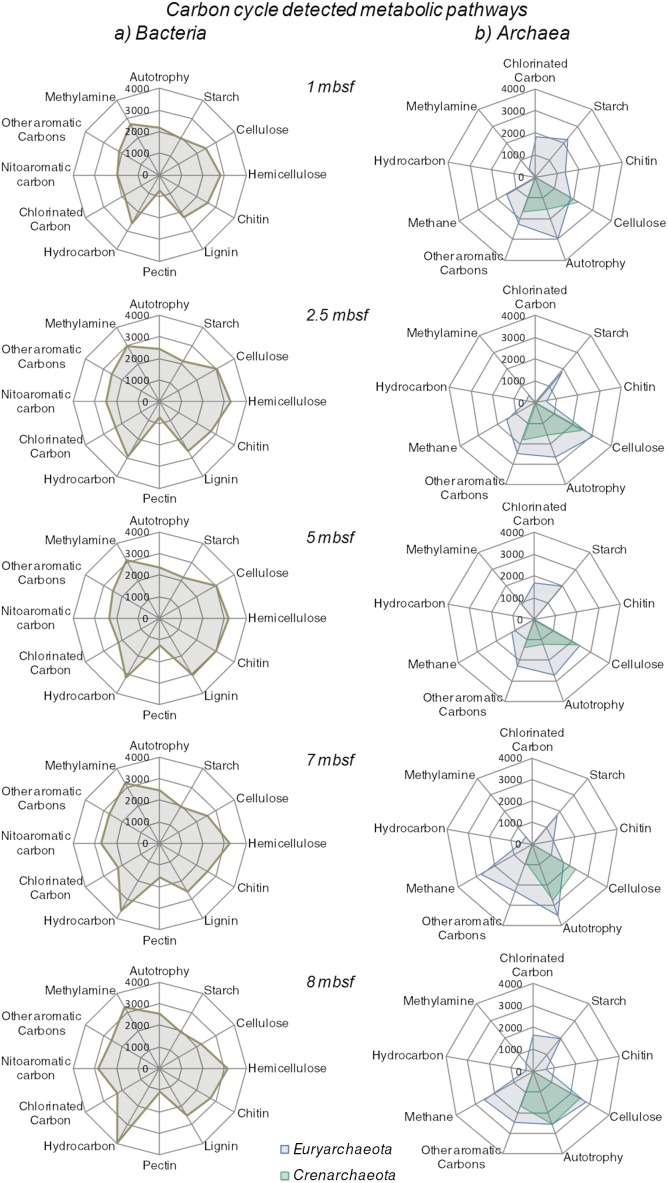
Carbon-cycling methabolic pathways detected by GeoChip. Carbon-cycling metabolic pathways identified for a) *Bacteria* and b) Archaeal *Euryarchaeota* (Blue) and *Crenarchaeota*-related (Green) lineages at different depths for BCK1 sediment core. Relative signal intensity was normalized by the number of the probes for each indicated metabolic pathway. List of targeted genes for each category are provided in Table S2.

In order to investigate the ecophysiology of the microbial community associated to subsurface Sonora Margin sediments, an array targeting functional genes was used for sediments collected at selected depths (1, 2.5, 5, 7 and 8 mbsf). The microarray results indicated a small but significant variation between the metabolic potential of microbial communities from each sediment horizon (ANOVA: F = 5.64, *P* = 0.002). Similarity percentages (SIMPER) and clustering analyses using Bray-Curtis similarity measure showed that the microbial communities associated with the 2.5 and 5 mbsf sediment horizons and the two deeper sediment horizons (7 and 8 mbsf) shared the greatest number of functional genes (93.3% and 91.92% similarity respectively), and that divergence between these metabolic potentials increased with sediment depth. These analyses indicated that this divergence was mainly due to the highest presence, in deepest sediment layer communities, of genes involved in hydrocarbon degradation (13% of variation) and in the upper sediment layers the predominance of genes involved in cellulose degradation (6.79% of variation, Figure 4). Using the taxonomic nature of the GeoChip probes [31], [32], putative metabolic functions were sorted according to specific taxonomic ranks: *Archaea* (3% of the total prokaryotic signal) or *Bacteria* (97%) super kingdoms and *Euryarchaeota* or *Crenarchaeota* phyla. *Crenarchaeota* phylum was recently revised to include only thermophilic lineages, excluding lineages such as MCG and MBGB [39]. However, GeoChip array was designed on the former phylogeny, thus the crenarchaeotal metabolic pathways detected in this study are likely to include MCG and MBGB lineages.

### Carbon metabolism

A large variety of bacterial genes for carbon utilization were identified (Figure 4). Genes coding for the RuBisCo, the propionyl-CoA/acetyl-CoA carboxylase (*ppc*), the ATP citrate lyase (*aclB*) and the carbon-monoxide dehydrogenase (CODH) were detected throughout the sediment core, indicating an autotrophic carbon fixation potential for both bacterial and archaeal lineages. Genes involved in heterotrophic metabolic pathways were also detected, indicating an important potential to transform a large variety of organic compounds. Bacterial genes associated with metabolic pathways for carbohydrates degradation (starch, cellulose, hemicellulose, chitin; lignin and pectin degradation), notably with extracellular enzyme genes, were detected in slightly higher proportion in the surface sediments. Hydrocarbon degradation pathway genes such as *chnA*, involved in ethylphenol and ethylbenzene catabolism, the *tut* operon, involved in toluene degradation and *alk* genes in the alkane degradation pathway [40] were also detected in increasing proportion with depth. The ability to degrade chlorinated, aromatic, polycyclic and xenobiotic compounds were also detected for bacteria, particularly with genes involved in the superpathway of aromatic compound degradation *via* 2-oxopent-4enoate and in the metacleavage of aromatic compounds [41]. Finally, the bacterial potential to use methylated amines was also identified throughout the sediment core. Archaeal metabolic genes for carbon utilization involved in carbohydrates and complex organic matter degradation as well as autotrophic metabolisms associated with *Euryarchaeota* and *Crenarchaeota*-related lineages were also detected. Finally, *mcrA* euryarchaeotal genes, involved in both methane production and anaerobic oxidation [42] were detected in increasing proportion with depth consistently with methane concentrations (Pearson correlation coefficient *r* = 0.832, *P* = 0.08; Figure 1e).

### Sulfate and Nitrogen metabolisms

**Figure 5:**
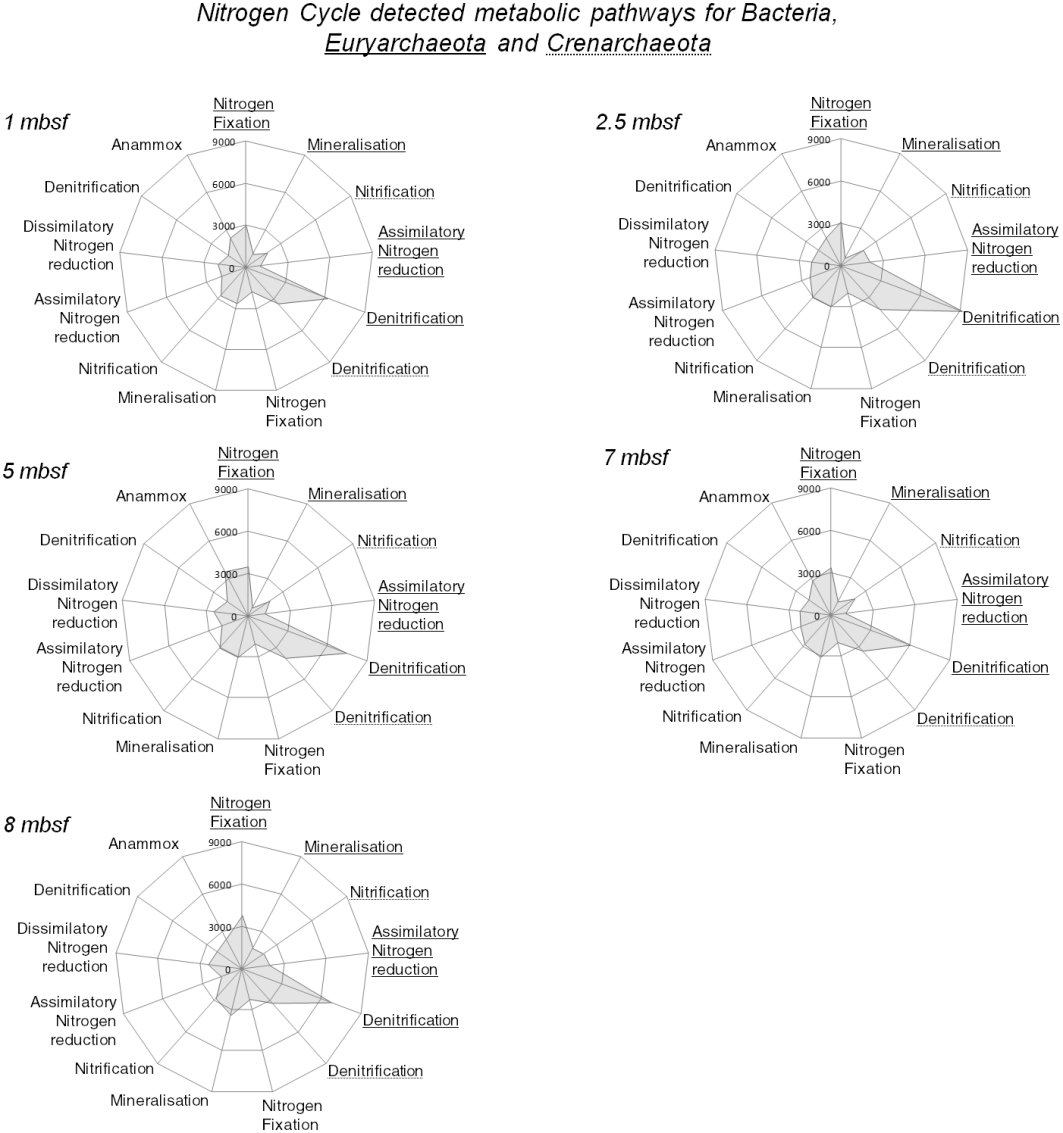
Nitrogen-cycling metabolic pathways identified at different depths for BCK1 sediment cores. Bacterial metabolic pathways are not underlined while *Euryarchaeota* and *Crenarchaeota*-related pathways are underlined with solid and dotted line respectively. Relative signal intensity was normalized by the number of the probes for each indicated metabolic pathway. List of targeted genes for each category are provided in Table S2.

The elevated ammonium concentrations measured in the sediments suggested that nitrogen cycle might be significant in the Sonora Margin sediments. Analyses of the functional gene array detected essential genes involved in the major pathways of the nitrogen cycle (Figure 5). Genes suggesting metabolic potentials for nitrogen fixation and mineralization (Glutamate dehydrogenase and urea amidohydrolase genes), allowing nitrogen input to the microbial ecosystem, were observed in both bacterial and euryarchaeotal lineages, while nitrification genes were detected in *Bacteria* and *Crenarchaeota*. Denitrification potential was identified in *Bacteria* and in higher proportion in *Archaea*. Hydrazine oxidoreductase genes involved in the anaerobic oxidation of ammonium (anammox) were also detected throughout the sediment core and in higher proportion (1.5 times) at 5 mbsf. Finally, genes involved in sulfate-reduction (*dsrAB, aprAB*) were identified throughout the sediments and in higher intensity at 1, 2.5 and 5 mbsf sediment horizons, which coincided with the sulfate-rich sediment layers (Figure 1).

## Discussion

### Microbial community structure

In this study, we document the taxonomic and functional diversity of the microbial community associated with subsurface sediments from a site adjacent (600 m) to cold seep sediment sites of the Sonora Margin [19], [20]. Although identical molecular methods were used in both studies, the microbial diversity associated with the subsurface sediments (0.5–9 mbsf) was different from the surface cold seeps (0–0.2 mbsf) of the Sonora Margin. For example, anaerobic methanotrophs and associated sulfate-reducing bacteria, observed in high concentrations in the cold seep surface sediments [19], [20] were not detected in subsurface sediments despite presence of a sulfate and methane transition zone. In contrast, the subsurface bacterial community was strongly dominated by members of *Chloroflexi* and candidate division phyla (JS1, OP8, etc.), and the major archaeal lineages detected were MCG, MBGB (also known as DSAG [43]) and MBGD. All these microbial populations have been frequently encountered in continental margin sediments and in the deep subsurface marine biosphere [9], [10], [13], but only in minor proportion in highly active ecosystems (hydrothermal vent, cold seeps) [20], [44] and in low carbon environments (open ocean sediments) [45]. Interestingly, no significant variation of the microbial community structure, excepted for the candidate division JS1, was detected throughout the sediment core, despite the presence of marked geochemical gradients (sulfate, methane). These results suggest that dominant microbial lineages were probably not directly involved in these biogeochemical cycles, as previously proposed for archaeal lineages [6], [37]. Overall, estimated cell abundance decreased with depth as commonly observed in marine sediments [3], [4]. In the Sonora Margin, elevated amounts of organic matter, derived from both marine production and continental inputs, sedimented in the seafloor with an estimated rate of 2 mm/y [46]. The accumulation of these sedimented particles led to an elevated sedimentary TOC content (3.5∼4%). Distance to land, geochemical gradients and organic carbon quality and abundance can control the microbial community structure and abundance in marine sediments [3], [7], [14], thus the high cellular abundance in the Sonora Margin sediments could be a consequence of the high concentrations of organic carbon. Elevated Q-PCR-based cell abundance estimations in the first meters of sediment could be due to higher concentrations of several electron acceptors (oxygen, nitrate, manganese and sulfate). Furthermore, significant correlations were found between TOC percentage and total *Bacteria*, *Chloroflexi*, candidate division JS1 and MBGD cell abundance estimations below 1.5 mbsf (Pearson correlation coefficients *r* = 0.58, 0.66, 0.75 and 0.66 respectively; *P*<0.04), which are consistent with reports of correlation between TOC and subsurface microbial biomass [7], [47]. Likewise, fluctuations below 3 mbsf of all microbial lineage cell abundances, appeared to be positively correlated with the local elementary composition of the sediments (Fe, Ti and Al, Pearson correlation coefficients *r*>0.67, *P*<0.04; Table S3). These results clearly indicate that in subsurface margin sediments microbial communities are influenced directly or indirectly by the geochemical composition of the sediments and suggest that the microbial abundance in margin ecosystems could be enhanced by the continental detrital inputs rather than by oceanic production, as indicated the correlations with terrigenous-derived metallic elements [48], [49]. This result is congruent with recent model calculations in subsurface sediments, indicating that buried organic carbon is sufficient to fuel microbial communities over turnover of millions of years [50].

### Organic matter degradation

Based on single cell genomics, it was recently proposed that archaeal MCG and MBGD lineages could degrade detrital organic matter [18]. Moreover, genes and transcripts, involved in anaerobic metabolism of amino acids, carbohydrates and lipids have been previously detected in the deep subsurface biosphere [16], [17]. However, it remains unclear how the microbial community is organized to degrade the detrital inputs and which microbial processes are involved. Although the GeoChip cannot be considered to be a comprehensive array with respect to marine sediment environments, it does contain an important number of relevant probes targeting genes involved in key biogeochemical cycles and represents an interesting approach to analyze the genomic potential in environments. The microbial metabolic potential analyzed using the GeoChip showed that the majority of the genes detected were related to various bacterial metabolic pathways for the transformation and the anaerobic degradation of simple and complex organic matter (Figure 1e). The high ammonium concentrations in these sediments could therefore be a consequence of the degradation of large amounts of organic matter by microbial communities associated to the Sonora Margin subsurface sediments. Genes associated with several metabolic pathways including extracellular and intracellular enzymes involved in the degradation and assimilation of decaying wood were detected, supporting the importance of subsurface microbial communities degrading organic matter such as plants and starch. For example, genes for transformation of lignin and complex organic aromatic substrates were also identified, notably involved in the superpathway of the aromatic compound cleavage, indicating that even the more recalcitrant wood particles could potentially be degraded by the bacterial community in the Sonora Margin (Figure 4a). This wood-based degradation metabolism appeared to be predominant in the upper sediment layers while hydrocarbon catabolism predominated the deeper sediment horizons. The Guaymas Basin sediments are well known to harbor various C_1_ to C_8_ hydrocarbon compounds such as ethane, butane, pentane and other alkanes [51]. Thus the bacterial community may be able to degrade this upward migrating organic carbon source as well as sedimented particles.

Other genes implicated in metabolic pathways for carbon assimilation have also been identified, indicating that different strategies for carbon assimilation occur amongst the different bacterial lineages (Figure 4a). For example, potential for degradation of chlorinated compounds was present, which is congruent with previous detection of dehalogenase enzymes and dehalogenation activities in similar deep biosphere sediments dominated by *Chloroflexi* lineages [52]. Degradation of chlorinated compounds derived from decaying marine phytoplankton pigments [53], suggests that in addition to terrestrial input the Sonora Margin bacterial community, (e.g. *Chloroflexi* members) could catabolize marine production and phytoplankton [54]. This metabolic specialization, which is energetically more favorable than sulfate reduction, may also explain the overall abundance of *Chloroflexi* representatives in marine sediments [45]. In addition, part of the bacterial community could also decompose decaying macrofauna with metabolic pathways involved in chitin and methylamine degradation. Finally, genetic potential for autotrophic metabolism was identified in both *Bacteria* and *Archaea* domains, suggesting that carbon dioxide could be either assimilated by specific microbial groups or that some subsurface microorganisms might be facultative heterotrophs, as previously suggested [5].

In contrast to bacterial lineages, the detected metabolic potential of *Archaea* appeared to be less diverse, maybe due to the more limited number of genes targeted by the GeoChip. Even if we could not exclude that our representation of the archaeal metabolic potential may be biased by unknown or non-targeted archaeal genes that escape to the microarray detection, various archaeal functional genes were identified. Crenarchaeotal-related lineages, likely including MCG and MBGB phyla, appeared to have the metabolic potential for complex organic carbon degradation (cellulose and aromatic polymers; Figure 4b). This result is supported by single cell MCG genomes [18] and distribution [37] suggesting heterotrophic metabolisms, possibly linked to aromatic compounds degradation [55]. Likewise, euryarchaeotal lineages, dominated by MBGD (95% based on Q-PCR estimations), appeared to have mainly the potential to degrade wood detrital polymers like starch, cellulose and aromatic compounds (Figure 4b). Hence, MBGD members could be anaerobic and heterotrophic degraders of complex organic matter, as previously suggested [18]. Resulting peptides from enzymatic degradations could be further assimilated by MBGD cells *via* peptidases and oligopeptide transporters, recently detected in their genome [18].

### Methane and Sulfate cycles

Interestingly, the low GeoChip signal intensity for the *mcrA* gene, a gene coding for an enzyme involved in production and anaerobic oxidation of methane [42] was correlated with methane concentrations and methanogenesis rates measured in the sediments (Figure 1). However, Q-PCR quantification and *mcrA* gene clone libraries (data not shown) only detected putative methane cycling *Archaea* related to *Methanococcoides* in sediments at 1 mbsf. Detection of these methanogens degrading noncompetitive substrates, such as methylated amines [56] is consistent with the presence of methanogenesis from dimethylamine and the detection of euryarchaeotal genes involved in methylamine degradation (Figure 4b). In deeper sediments with low methanogenesis rates (10–100 fold lower than in cold seeps [57]), relative abundances of known methanogens were probably below the PCR and Q-PCR detection limits (<1000 16S rRNA gene copy per gram of sediment) or escape amplification due to primer deficiencies [13]. As suggested by the changing δ13-CH_4_ signature, anaerobic methanotrophs could also be present in extremely low abundance or with altered key genes that would escape molecular detection [58]. These methanotrophs might be coupled directly or indirectly with sulfate-reducing *Deltaproteobacteria*, detected between 4 and 7 mbsf by 16S rRNA libraries and *dsrAB AprAB* GeoChip probes and thereby, lead to the formation of the SMTZ in these sediments (Figure 1).

### Nitrogen cycle

Key bacterial metabolic genes involved in the nitrogen cycle were also detected with the microarray approach in the Sonora Margin sediments (Figure 5). In addition to nitrogen fixation, denitrification and anammox by bacterial communities, *Euryarchaeota* showed genetic potential for nitrogen fixation. Nitrogen assimilation is an important metabolic process for deep subsurface sediment microbial communities [5] and various members of the *Euryarchaeota* such as methanogenic lineages [59], [60], ANME-2 [61] and ANME-1 [62] were previously found to anaerobically fix nitrogen. The detection of euryarchaeotal nitrogen fixation genes in our results suggested that members of MBGD, representing 95% of the *Euryarchaeota* could also be diazotrophic *Archaea*. Nitrification (ammonium oxidation) genes (*amoA*) were identified as a potential metabolism in crenarchaeotal-related lineages. Although ammonium, a potential electron donor, is abundant in the Sonora Margin sediments, probably due to organic matter microbial degradation, the presence of such oxygenase enzymes in this anoxic environment remains enigmatic [47], [63]. It was therefore suggested that ammonium oxidation could be performed using an alternative electron acceptor [47] or that *amo* genes in anoxic environments could have an alternative function [64]. Consistently with the detection of *nar* transcripts in deep marine sediments [16], archaeal and bacterial denitrification genes were present throughout the sediment core, which could contribute to the elevated ammonium concentrations. Anaerobic ammonium oxidation was previously suggested for the nitrate origin in the deepest sediments as it could potentially be produced as a by-product of the process [16]. This would be supported by the detection of the *hzo* genes by the GeoChip probes, as well as the previously report of anammox process in the Sonora Margin sediments [65].

### Conclusion

This study clearly indicated that Sonora Margin sub-surface sediment microbial communities, probably controlled by terrigeneous inputs, are composed of deep biosphere-related microorganisms, distinct of the Sonora Margin surface cold seep communities. Consistently, genetic potentials for the catabolism of complex organic matters (decaying wood, macrofauna, phytoplankton and hydrocarbon) were identified, suggesting that various heterotrophic strategies occur amongst sedimentary microbial communities. Further specific measurements of rates of degradation these different substrates could confirm these results and lead to a better understanding of the biochemical processes driving subseafloor microbial communities.

## Supporting Information

Figure S1 Geochemical depth profiles of: total iron, aluminum, potassium, manganese, total sulfur, silica and titanium concentrations in the unfiltered pore waters of the BCK1 core. The blue shade represent important changes in elemental composition profiles.
(TIF)

Figure S2 Rarefaction curves for A) archaeal and B) Bacterial 16S rRNA gene libraries.
(TIF)

Figure S3 Maximum Likelihood phylogenetic tree of the archaeal 16S cDNA sequences amplified from sections 1, 4, 5, 7 and 8 mbsf (labeled S1, S4, S5, S7 and S8 respectively) of the BCK1 sediment core. Phylogenetic tree was performed using RAxML 7.2.8. and GTRCAT model approximation with 1000 replicates. Only bootstrap values up to 70% are shown. Only one representative sequence (>97% identical) per sediment horizon is shown. Number in brackets shown the number of clones analyzed from RNA clone libraries. MBG-D/B, Marine Benthic Group D/B; TMEG, Terrestrial Miscellaneous Euryarcheotal Group; MCG, Miscellaneous Crenarchaeotal Group; MHVG, Marine Hydrothermal Vent Group; SAGMEG, South Africa Gold Mine Euryarchaeotal Group.
(TIF)

Figure S4 Maximum Likelihood phylogenetic tree of the bacterial 16S cDNA sequences amplified from sections 1, 4, 5, 7 and 8 mbsf (labeled S1, S4, S5, S7 and S8 respectively) of the BCK1 sediment core. Phylogenetic tree was performed using RAxML 7.2.8. and GTRCAT model approximation with 1000 replicates. Only bootstrap values up to 70% are shown. Only one representative sequence (>97% identical) per sediment horizon is shown. Number in brackets shown the number of clones analyzed from RNA clone libraries.
(TIF)

Table S1 Primer sets and annealing temperatures used for real-time PCR of 16S rRNA gene.
(DOC)

Table S2 Details of GeoChip-targeted genes, related proteins and processes corresponding to each identified metabolic pathways.
(DOC)

Table S3 Correlation statistical tests and associated P values for microbial lineages and elementary composition of the sediment pore-waters.
(DOC)

Methods S1 Detailed methods for methanogenesis activity measurements, gene library constructions and phylogenetic affiliations, Q-PCR and ARISA experiments.
(DOCX)
