# Supplementary figures and images for: Phylogenetic and Functional Diversity of Microbial Communities Associated with Subsurface Sediments of the Sonora Margin, Guaymas Basin
Source: PLoS One. 2014 Aug 6;9(8):e104427. doi: 10.1371/journal.pone.0104427 (PMC4123917; doi:10.1371/journal.pone.0104427)

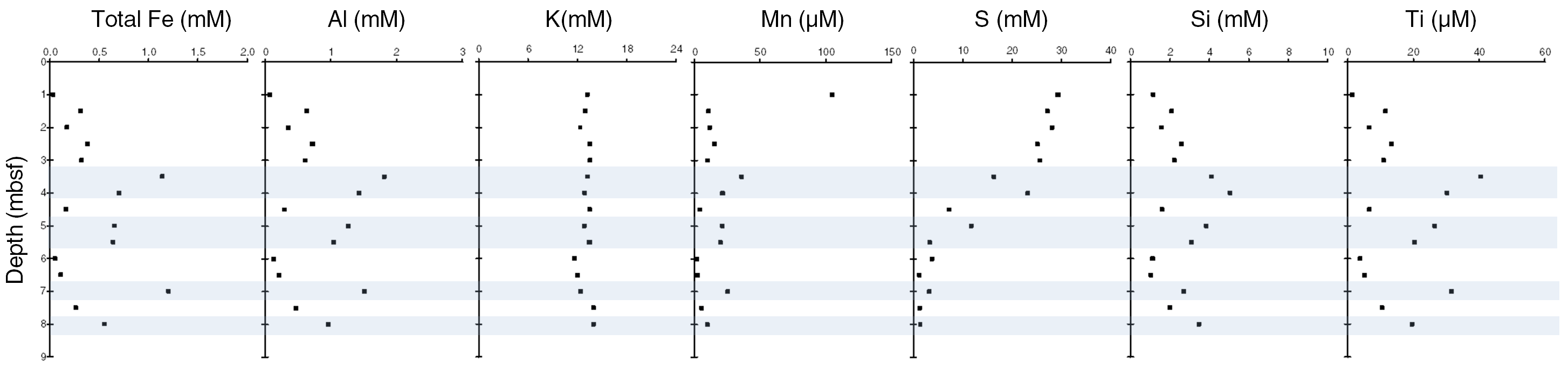

Supplement: Figure S1 — Geochemical depth profiles of: total iron, aluminum, potassium, manganese, total sulfur, silica and titanium concentrations in the unfiltered pore waters of the BCK1 core. The blue shade represent important changes in elemental composition profiles. (TIF) [file pone.0104427.s001.tif]

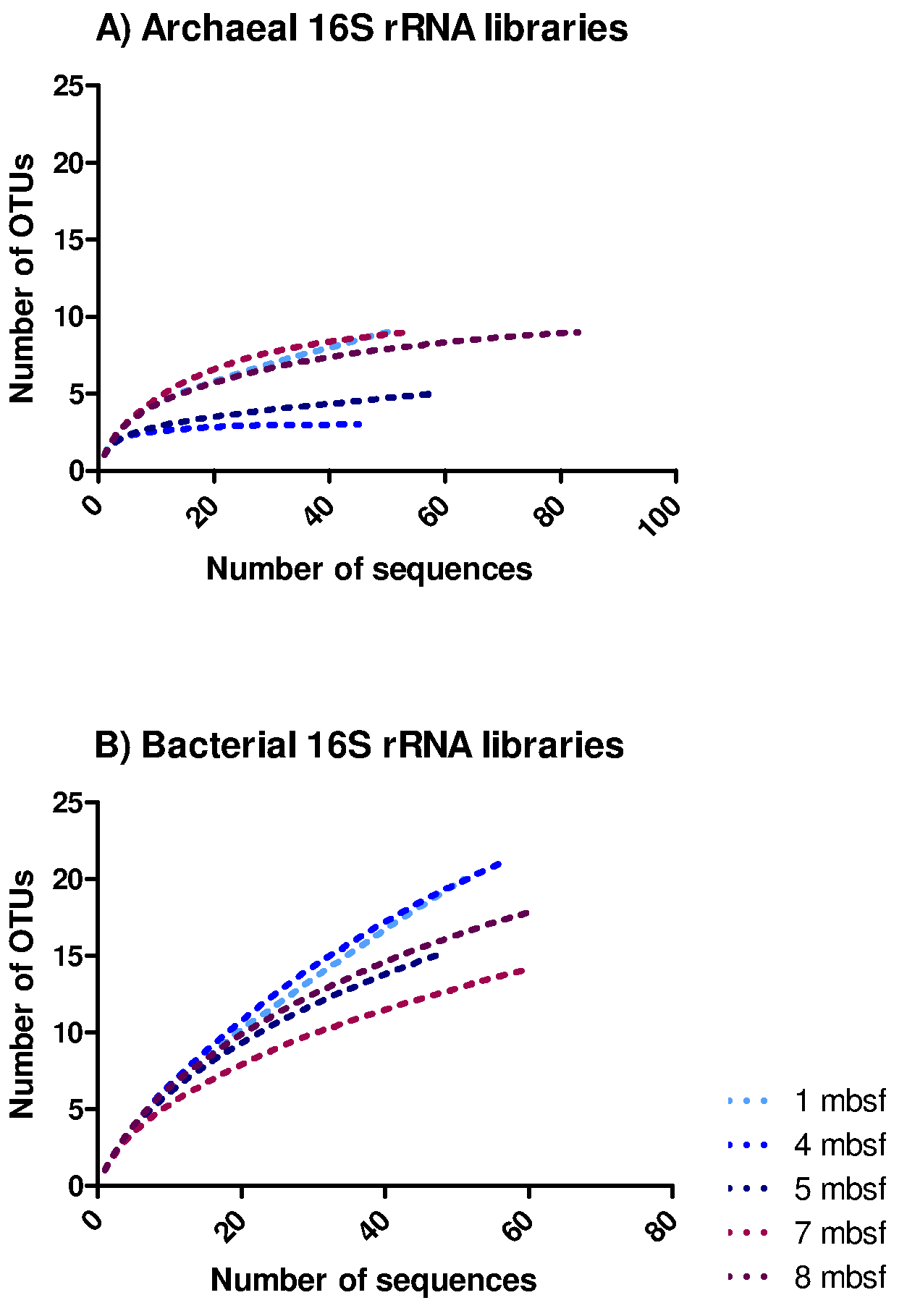

Supplement: Figure S2 — Rarefaction curves for A) archaeal and B) Bacterial 16S rRNA gene libraries. (TIF) [file pone.0104427.s002.tif]

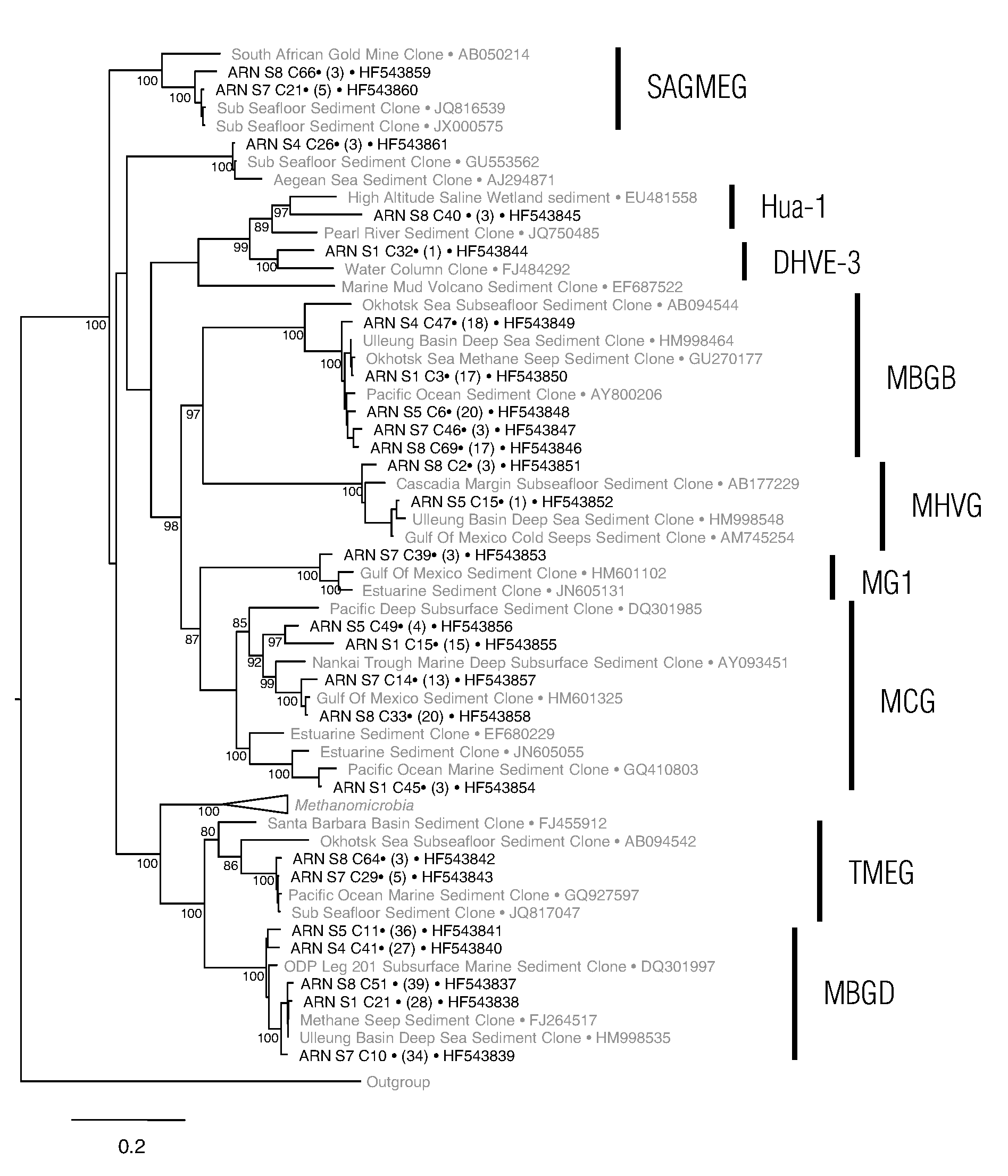

Supplement: Figure S3 — Maximum Likelihood phylogenetic tree of the archaeal 16S cDNA sequences amplified from sections 1, 4, 5, 7 and 8 mbsf (labeled S1, S4, S5, S7 and S8 respectively) of the BCK1 sediment core. Phylogenetic tree was performed using RAxML 7.2.8. and GTRCAT model approximation with 1000 replicates. Only bootstrap values up to 70% are shown. Only one representative sequence (>97% identical) per sediment horizon is shown. Number in brackets shown the number of clones analyzed from RNA clone libraries. MBG-D/B, Marine Benthic Group D/B; TMEG, Terrestrial Miscellaneous Euryarcheotal Group; MCG, Miscellaneous Crenarchaeotal Group; MHVG, Marine Hydrothermal Vent Group; SAGMEG, South Africa Gold Mine Euryarchaeotal Group. (TIF) [file pone.0104427.s003.tif]

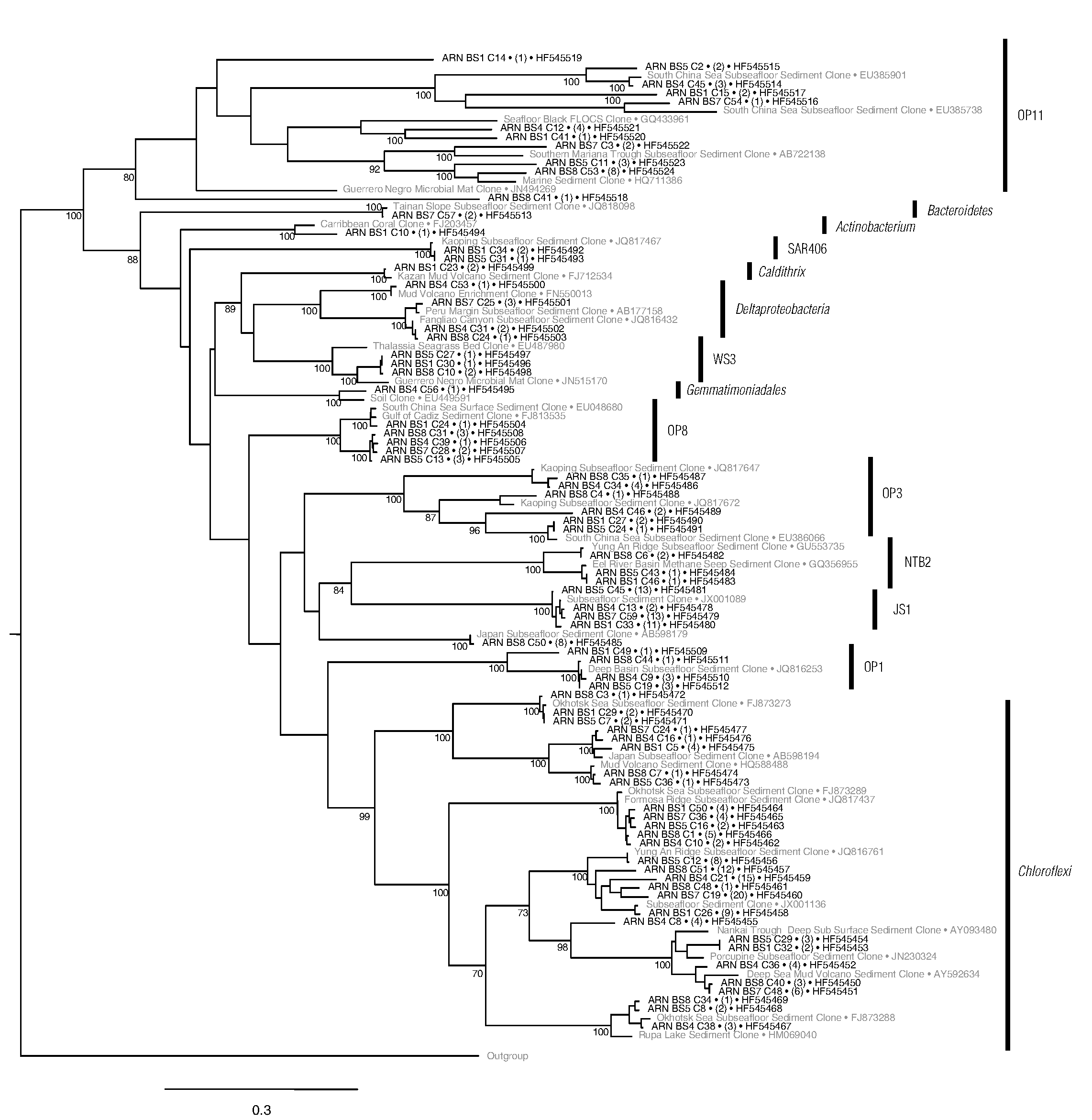

Supplement: Figure S4 — Maximum Likelihood phylogenetic tree of the bacterial 16S cDNA sequences amplified from sections 1, 4, 5, 7 and 8 mbsf (labeled S1, S4, S5, S7 and S8 respectively) of the BCK1 sediment core. Phylogenetic tree was performed using RAxML 7.2.8. and GTRCAT model approximation with 1000 replicates. Only bootstrap values up to 70% are shown. Only one representative sequence (>97% identical) per sediment horizon is shown. Number in brackets shown the number of clones analyzed from RNA clone libraries. (TIF) [file pone.0104427.s004.tif]
