# Supplementary material for: Phylogenetic and Functional Diversity of Microbial Communities Associated with Subsurface Sediments of the Sonora Margin, Guaymas Basin
Source: PLoS One. 2014 Aug 6;9(8):e104427. doi: 10.1371/journal.pone.0104427 (PMC4123917; doi:10.1371/journal.pone.0104427)
Supplement: Table S1 — Primer sets and annealing temperatures used for real-time PCR of 16S rRNA gene. (DOC) [file pone.0104427.s005.doc]

**Table 1. PCR primers used for PCR, and real-time PCR of 16S rR**NA genes

| **Name** | **Function** | **Target group** | **Sequence (5' - 3')** | **Amplicon size (bp)** | **Annealing Temp. (°C)** | **Primer conc. (mM)** | **Ref.** |
| --- | --- | --- | --- | --- | --- | --- | --- |
| **A8F**  **A915R** | Cloning  Sequencing | Most of *Archaea* | CGG-TTG-ATC-CTG-CCG-GA  GTG-CTC-CCC-CGC-CAA-TTC-CT | 900 | 57 | 0.4 |  |
| **E338F**  **U1407R** | Cloning  Sequencing | Most of *Bacteria* | ACT-CCT-ACG-GGA-GGC-AGC  GAC-GGG-CGG-TGW-GTR-CAA | 1000 | 54 | 0.4 |  |
| **Mcc495F**  **McocR** | Q-PCR | *Methanococcales* | TAA-GGG-CTG-GGC-AAG-T  GGA-TCA-ACA-CAT-TTC-ACC-GC | 186 | 61 | 1.1 |  |
| **MBT857F**  **MBT1196R** | Q-PCR | *Methanobacteriales* | CGW-AGG-GAA-GCT-GTT-AAG-T  TAC-CGT-CGT-CCA-CTC-CTT | 343 | 60 | 0.3 |  |
| **MmicF**  **MmicR** | Q-PCR | *Methanomicrobiales* | GTG-ATA-AGG-GAA-CCY-CGA-G  GCT-ACG-RAC-GCT-TTA-AGC-C | 126 | 61 | 0.9 |  |
| **MsarF**  **Msar2R** | Q-PCR | *Methanosarcinales* | GTA-CCT-ACT-AGC-CDA-CRA-CGG  GCT-ACR-GAC-CCT-TTA-GAC-CC | 244 | 64 | 0.9 | ([Vigneron et al 2013](#_ENREF_7)) |
| **MpyrF**  **MpyrR** | Q-PCR | *Methanopyrales* | GGC-TAA-TCC-CCG-ATA-GGC  CTT-GTC-TCA-GTC-CCC-GTC-TC | 151 | 61 | 0.8 | ([Vigneron et al 2013](#_ENREF_7)) |
| **ANME1F**  **ANME1R** | Q-PCR | ANME1 | GCT-TTC-AGG-GAA-TAC-TGC  TCG-CAG-TAA-TGC-CAA-CAC | 200 | 60 | 1.1 |  |
| **ANME2aF**  **ANME2aR** | Q-PCR | ANME2a | ACG-GAT-ACG-GGT-TGT-GAG-AG  CTT-GTC-TCA-GTC-CCC-GTC-TC | 151 | 60 | 1.1 | ([Vigneron et al 2013](#_ENREF_7)) |
| **ANME2cR**  **ANME2cF** | Q-PCR | ANME2c | TCC-TCT-GGG-AAA-TCT-GGT-TG  TCG-TTT-ACG-GCT-GGG-ACT-AC | 224 | 60 | 1.1 | ([Vigneron et al 2013](#_ENREF_7)) |
| **ANME3F**  **ANME3R** | Q-PCR | ANME3 | GGA-TTG-GCA-TAA-CAC-CGG  TAT-GCT-GGC-ACT-CAG-TGT-CC | 234 | 60 | 1.1 | ([Vigneron et al 2013](#_ENREF_7)) |
| **MCG-528F**  **MCG-780R** | Q-PCR | MCG | CGG-TAA-TAC-CAG-CTC-TCC-GAG  TTC-GCT-CCC-CCA-GCT-T | 268 | 60 | 0.9 | This study |
| **MBGB-302F**  **MBGB-423R** | Q-PCR | MBGB | CGA-TAA-TCG-ATA-GGG-GCC-GT  TGG-GTA-ACC-CCG-TCA-CA | 138 | 60 | 1.1 | This study |
| **MBGD-345F**  **MBGD-490R** | Q-PCR | MBGD | ATA-TCT-GAG-ACA-CGA-TAT-CRG-G  CAC-CAC-TTG-AGC-TGC-AGG-TA | 227 | 60 | 1.1 | This study |
| **JSChlo-519f**  **JSChlo-655r** | Q-PCR | Chloroflexi/ Cand. Div. JS1 | CAG-CAG-CCG-CGG-TAA-YAC  ACC-GGG-AAT-TCC-ACY-TYC-CT | 156 | 60 | 0.9 |  |
| **JS1-318**  **JS1-410** | Q-PCR | Cand. Div. JS1 | TTG-AGG-TTA-GAA-GAG-GAA-AGT  GAG-ATA-GAC-CAG-AAA-GCC-GC | 112 | 60 | 1 |  |
| **DSS-314F**  **DSS-474R** | Q-PCR | *Desulfosarcina/coccus* | ACT-TGA-GTA-TGG-GAG-AGG-GAA-G  ACC-TAG-TGT-TCA-CCG-TTT-ACT-GC | 180 | 60 | 1 |  |
| **DBB-314F**  **DBB-474R** | Q-PCR | *Desulfobulbus* | GCT-TGA-GTA-TGG-GAG-AGG-GAA-G  CAC-CTA-GTT-CTC-ATC-GTT-TAC-AGC | 180 | 60 | 1 |  |
| **SRB2-314F**  **SRB2-474R** | Q-PCR | SEEP SRB2 | ACT-TGA-GTA-CCG-GAG-AGG-GA  CCT-AGT-GCC-CAT-CGT-TTA-GG | 180 | 60 | 1 |  |
| **ARC787F**  **ARC1059R** | Q-PCR | *Archaea* | ATT-AGA-TAC-CCS-BGT-AGT-CC  GCC-ATG-CAC-CWC-CTC-T | 273 | 60 | 0.5 |  |
| **BACT1369F**  **BACT1492R** | Q-PCR | *Bacteria* | CGG-TGA-ATA-CGT-TCY-CGG  GGW-TAC-CTT-GTT-ACG-ACT-T | 142 | 58 | 0.6 |  |

**Blazejak, A., and Schippers, A. (2010) High abundance of JS-1-and Chloroflexi-related Bacteria in deeply buried marine sediments revealed by quantitative, real-time PCR. *FEMS Microbiol Ecol* 72: 198-207.**

**Cambon-Bonavita, M.A., Nadalig, T., Roussel, E., Delage, E., Duperron, S., Caprais, J.C. et al. (2009) Diversity and distribution of methane-oxidizing microbial communities associated with different faunal assemblages in a giant pockmark of the Gabon continental margin. *Deep-Sea Research Part Ii-Topical Studies in Oceanography* 56: 2248-2258.**

**Kubo, K., Lloyd, K.G., J, F.B., Amann, R., Teske, A., and Knittel, K. (2012) Archaea of the Miscellaneous Crenarchaeotal Group are abundant, diverse and widespread in marine sediments. *ISME J*.**

**Lloyd, K.G., Alperin, M.J., and Teske, A. (2011) Environmental evidence for net methane production and oxidation in putative ANaerobic MEthanotrophic (ANME) archaea. *Environ Microbiol* 13: 2548-2564.**

**Suzuki, M.T., Taylor, L.T., and DeLong, E.F. (2000) Quantitative analysis of small-subunit rRNA genes in mixed microbial populations via 5'-nuclease assays. *Appl Environ Microbiol* 66: 4605-4614.**

**Teske, A., and Sorensen, K.B. (2008) Uncultured archaea in deep marine subsurface sediments: have we caught them all? *Isme Journal* 2: 3-18.**

**Vigneron, A., Cruaud, P., Pignet, P., Caprais, J.-C., Cambon-Bonavita, M.-A., Godfroy, A., and Toffin, L. (2013) Archaeal and anaerobic methane oxidizer communities in the Sonora Margin cold seeps, Guaymas Basin (Gulf of California). *ISME J*.**

**Vigneron, A., Cruaud, P., Pignet, P., Caprais, J.-C., Gayet, N., Cambon-Bonavita, M.-A. et al. (2014) Bacterial communities and syntrophic associations involved in anaerobic oxidation of methane process of the Sonora Margin cold seeps, Guaymas Basin. *Environ Microbiol*: n/a-n/a.**

**Yu, Y., Lee, C., Kim, J., and Hwang, S. (2005) Group-specific primer and probe sets to detect methanogenic communities using quantitative real-time polymerase chain reaction. *Biotechnology and Bioengineering* 89: 670-679.**
