# Supplementary material for: Phylogenetic and Functional Diversity of Microbial Communities Associated with Subsurface Sediments of the Sonora Margin, Guaymas Basin
Source: PLoS One. 2014 Aug 6;9(8):e104427. doi: 10.1371/journal.pone.0104427 (PMC4123917; doi:10.1371/journal.pone.0104427)
Supplement: Table S2 — Details of GeoChip-targeted genes, related proteins and processes corresponding to each identified metabolic pathways. (DOC) [file pone.0104427.s006.doc]

**Table 2**. Details of targeted genes, related proteins and processes corresponding to each identified metabolic pathways.

| Carbon cycling | Autotrophy | Calvin–Benson–Bassham cycle | RubisCo |
| --- | --- | --- | --- |
|  |  | 3-hydroxypropionate/malyl-CoA cycle | *pcc* |
|  |  | Reductive acetyl-CoA pathway | *aclB* and *CODH* |
|  | Carbon carbohydrates degradation | Starch degradation | Alpha-amylase (*amyA*), amylopullulanase (*amyX*, *apu*), cyclomaltodextrin dextrin-hydrolase (*cda*), glucoamylase, isopullulanase, neopullulanase II (*nplT*), and pullulanase (*pulA*) genes |
|  |  | Cellulose degradation | Cellobiose dehydrogenase, cellobiase (*bgl*), endoglucanase (*egl*), and exoglucanase genes |
|  |  | Hemicellulose degradation | Arabinofuranosidase, mannanase, xylose isomerase (*xylA*), and xylanase genes |
|  |  | Chitin degradation | Acetylglucosaminidase, endochitinase, and exochitinase genes |
|  |  | Pectin degradation | Pectinase genes |
|  | Other carbon degradation | Hydrocarbon degradation | *ChnA/B/C,* *alkA/B/J/K/H*, *tutF/D/G* genes |
|  |  | Chlorinated carbon degradation | *Rd, tftH, cbdA, tfdA* and dehalogenase (*dehH*) genes |
|  |  | Nitoaromatic carbon degradation | *pnbA, nmoA, nhh*, and xenobiotic reductase (*nitro*) genes |
|  |  | Other aromatic carbon | 54 various genes including *xylG/C/XY/J/F/L, pimF,mdlA/B/C/D.* |
|  |  | Methylamine | *dmsA* |
|  | Methane | Methane production | *mcrA* |
| Nitrogen cycling | | Nitrogen fixation | Nitrogenase reductase genes (*nifH*) |
|  |  | Mineralisation | Glutamate dehydrogenase (*gdh*) and urea amidohydrolase (*ureC*) |
|  |  | Nitrification | Ammonia monooxygenase (*amoA*) and hydroxylamine oxidase (*hao*) |
|  |  | Assimilatory nitrogen reduction | Assimilatory nitrate reductase (*nasA*) and nitrite reductase (*nir, nirA/B*) |
|  |  | Dissimilatory nitrogen reduction | Nitrate reductase (*nirS*) and nitrite reductase (*nrfA*) |
|  |  | Denitrification | Nitrate reductase (*narG*),nitrite reductase (*nirK, nirS*), nitric oxide reductase (*norB*) and nitrous oxide reductase (*nosZ*) |
|  |  | Anammox | Hydrazine oxidoreductase (*hzo*) |
