# Supplementary material for: Phylogenetic and Functional Diversity of Microbial Communities Associated with Subsurface Sediments of the Sonora Margin, Guaymas Basin
Source: PLoS One. 2014 Aug 6;9(8):e104427. doi: 10.1371/journal.pone.0104427 (PMC4123917; doi:10.1371/journal.pone.0104427)
Supplement: Table S3 — Correlation statistical tests and associated P values for microbial lineages and elementary composition of the sediment pore-waters. (DOC) [file pone.0104427.s007.doc]

| **Pearson Correlation test** | ***Archaea*** | **MBGD** | **MBGB** | **MCG** | ***Bacteria*** | ***Chloroflexi*** | **Cand. Div. JS1** |
| --- | --- | --- | --- | --- | --- | --- | --- |
| **Fe** | r= 0.8 p=0.004 | r=0.651 p=0.04 | r=0.736 p=0.01 | r=0.670 p=0.03 | r=0.736 p=0.01 | r=0.703 p=0.02 | r=0.690 p=0.03 |
| **Ti** | r=0.747 p=0.01 | r=0.700 p=0.02 | r=0.769 p=0.01 | r=0.671 p=0.04 | r=0.788 p=0.006 | r=0.758 p=0.01 | r=0.752 p=0.01 |
| **Al** | r=0.748 p=0.01 | r=0.680 p=0.03 | r=0.761 p=0.01 | r=0.712 p=0.03 | r=0.763 p=0.01 | r=0.729 p=0.02 | r=0.724 p=0.02 |

**Table 3 Correlation statistical tests and associated P values for microbial lineages and elementary composition of the sediment pore-waters.**
