# Supplementary material for: Phylogenetic and Functional Diversity of Microbial Communities Associated with Subsurface Sediments of the Sonora Margin, Guaymas Basin
Source: PLoS One. 2014 Aug 6;9(8):e104427. doi: 10.1371/journal.pone.0104427 (PMC4123917; doi:10.1371/journal.pone.0104427)
Supplement: Methods S1 — Detailed methods for methanogenesis activity measurements, gene library constructions and phylogenetic affiliations, Q-PCR and ARISA experiments. (DOCX) [file pone.0104427.s008.docx]

**Supplementary Methods**

Methanogenesis activity measurements

Potential rates of methanogenesis were monitored on anaerobically stored sub-samples using 14C labeled substrates, three months post-cruise, at Cardiff University, UK. The 5 mL syringes were injected with radiotracers (14C-bicarbonate [19 µL containing 74 kBq], 14C-acetate [19 µL containing 397 kBq], 14C-di-methylamine [19 µL containing 176 kBq]) and incubated for 7 – 14 days (bicarbonate), 2 days (acetate) and 3 – 6 days (di-methylamine) at close to in situ temperatures (4°C). Activity measurements were terminated by expulsion of the sample into a 30 mL vial containing 7 mL of 1M NaOH followed by vigorous shaking to disperse the sediment plugs. Produced methane was measured by flushing the vial headspace, using N2/O2 (95:5), across CuO at 900°C and collecting the 14CO2 in series of three scintillation vials containing 10 mL of Hi-Safe 3 scintillation cocktail (Canberrra Packard, UK) containing 7% b-phenethylamine. Methanogenesis rates from 14C-acetate, 14C-di-methylamine and 14C-bicarbonate were calculated based on the proportion of labeled gas produced from the 14C-substrate, the incubation time period, an assumed sediment porosity of 70% and the cold pool size of the substrate. For di-methylamine no cold pool was detected and the di-methylamine added as the label (2.22 x 104 DPM = 1 nmol) was used as the pool size. Activity rates were expressed in pmol/cm3/day to be compared with other studies.

Gene libraries

1, 4, 5,7 and 8 mbsf sediment layers were selected as representative horizons for the microbial community analyses. Gene libraries from 16S rRNA templates were constructed from pooled and gel purified triplicate reactions in TOPO® XL PCR Cloning Kit (Invitrogen, Carlsbad, CA, USA) according to manufacturer's recommendations. Sequencing was done on an ABI3730xl - genetic Analyzer using M13 universal primers by GATC Biotech (Germany). Sequences were analyzed using NCBI BLAST search program GenBank and aligned with the closest representative sequences using MAFFT 6.903 ([Katoh et al., 2005](#_ENREF_5)). Presence of chimera was checked manually. Phylogenetic trees were estimated with maximum likelihood method, using RAxML 7.2.8 ([Stamatakis, 2006](#_ENREF_9)) on the CIPRES Science Gateway ([Miller et al., 2011](#_ENREF_7)). GTRCAT approximation of models was used for ML bootstrapping (1000 replicates). Rarefaction curves and diversity indices were performed using MOTHUR for all libraries ([Schloss et al., 2009](#_ENREF_8)).

Microbial community ARISA fingerprinting

An Automated method of Ribosomal Intergenic Spacer Analysis (ARISA) was carried out on archaeal and bacterial DNA 16S-23S intergenic spacer regions using A915f-A71r(23S) and ITSf-ITSr primers respectively ([Casamayor et al., 2002](#_ENREF_2); [Cardinale et al., 2004](#_ENREF_1)) to monitor the microbial community structure with depth (every 50 cm from 1 mbsf to 9 mbsf). PCR conditions were as follows: denaturation at 95°C for 30 s, annealing at 55°C for 30 s and extension for 1 min 20 s at 72°C for 35 cycles, followed by a final extension step at 72°C for 15 min Amplicons were analyzed using DNA 7500® Chip on an Agilent 2100 Bioanalyzer® (Agilent Technology, Santa Clara, USA) according to manufacturer's protocol. Data were recovered using 2100 Expert® software (Agilent Technology), then normalized by the raw signal intensity for each sample and statistically analyzed using Past® software ([Hammer et al., 2001](#_ENREF_4)). Non-metric multidimensional scaling (NMDS) and clustering analyzes were based on Bray-Curtis similarity measure.

Real-time (q)PCR

Triplicate quantitative measurements were monitored on every sample with a Step One instrument (Applied Biosystems, Foster City, CA, USA) in a final volume of 25 µL by using Perfecta® SYBR® Green SuperMix ROX (Quanta Bioscience), template and optimal primer concentrations (Supplementary Table 1) according to manufacturer instructions. Amplifications of crude DNA samples indicated presence of inhibitors in the samples, then Q-PCR reactions were performed on purified samples. Purified samples were diluted until Cp decreased log-linearly with dilutions, indicating the absence of inhibition. Primer sets, listed in Table 1, targeting *Archaea* and *Bacteria* previously found in the Sonora Margin sediments were completed with new primer sets for uncultivated *Archaea* of the MBGB and MBGD designed using ARB package ([Ludwig et al., 2004](#_ENREF_6)) and web-based application Primaclade ([Gadberry et al., 2005](#_ENREF_3)). For calibrations, environmental 16S rRNA gene representatives for the quantified uncultured microorganisms were inserted into plasmids. Standard curves were obtained by triplicate amplifications of plasmid dilutions ranging from 10^3^ to 10^8^ copies/µL. Resulted R^2^ of standard curves were up to 0.997 and efficiency of the reaction above 94 %. Primer sets specificity was confirmed by amplification products sequencing. Q-PCR results were expressed in 16S rRNA gene copy number per gram of sediment. Correlation factors with geochemical parameters and statistical tests on the microbial distribution were achieved using Graph Pad Prism Software.

**References**

Cardinale, M., Brusetti, L., Quatrini, P., Borin, S., Puglia, A.M., Rizzi, A. et al. (2004) Comparison of different primer sets for use in automated ribosomal intergenic spacer analysis of complex bacterial communities. *Appl Environ Microbiol* **70**: 6147-6156.

Casamayor, E.O., Massana, R., Benlloch, S., Ovreas, L., Diez, B., Goddard, V.J. et al. (2002) Changes in archaeal, bacterial and eukaryal assemblages along a salinity gradient by comparison of genetic fingerprinting methods in a multipond solar saltern. *Environ Microbiol* **4**: 338-348.

Gadberry, M.D., Malcomber, S.T., Doust, A.N., and Kellogg, E.A. (2005) Primaclade--a flexible tool to find conserved PCR primers across multiple species. *Bioinformatics* **21**: 1263-1264.

Hammer, Ø., DA, T.H., and PD, R. (2001) PAST: Paleontological Statistics Software Package for Education and Data Analysis. *Palaeontologia Electronica* **4**.

Katoh, K., Kuma, K., Toh, H., and Miyata, T. (2005) MAFFT version 5: improvement in accuracy of multiple sequence alignment. *Nucleic Acids Research* **33**: 511-518.

Ludwig, W., Strunk, O., Westram, R., Richter, L., Meier, H., Yadhukumar et al. (2004) ARB: a software environment for sequence data. *Nucleic Acids Res* **32**: 1363-1371.

Miller, M.A., Pfeiffer, W., and Schwartz, T. (2011) The CIPRES science gateway: a community resource for phylogenetic analyses. In *Proceedings of the 2011 TeraGrid Conference: Extreme Digital Discovery*. Salt Lake City, Utah: ACM, pp. 1-8.

Schloss, P.D., Westcott, S.L., Ryabin, T., Hall, J.R., Hartmann, M., Hollister, E.B. et al. (2009) Introducing mothur: Open-Source, Platform-Independent, Community-Supported Software for Describing and Comparing Microbial Communities. *Appl Environ Microbiol* **75**: 7537-7541.

Stamatakis, A. (2006) RAxML-VI-HPC: maximum likelihood-based phylogenetic analyses with thousands of taxa and mixed models. *Bioinformatics* **22**: 2688-2690.
